# Impact of the Spanish Smoking Law on Exposure to Secondhand Smoke in Offices and Hospitality Venues: Before-and-After Study

**DOI:** 10.1289/ehp.11845

**Published:** 2008-09-19

**Authors:** Manel Nebot, Maria J. López, Carles Ariza, Mónica Pérez-Ríos, Marcela Fu, Anna Schiaffino, Gloria Muñoz, Esteve Saltó, Esteve Fernández

**Affiliations:** 1 Public Health Agency, Barcelona, Spain; 2 Department of Experimental and Health Sciences, Universitat Pompeu Fabra, Barcelona, Spain; 3 Centro de Investigación Biomédica en Red de Epidemiología y Salud Pública (CIBER ESP), Spain; 4 PhD Programme in Public Health and Methodology of Biomedical Research, Universitat Autònoma de Barcelona, Barcelona, Spain; 5 Department of Preventive Medicine; 6 Department of Health, Xunta de Galicia, Santiago de Compostela, Spain; 7 Catalan Institute of Oncology and Institute of Biomedical Research of Bellvitge, L’Hospitalet de Llobregat, Spain; 8 Municipal Health Institute, City Council of Terrassa, Terrassa, Spain; 9 Department of Health, Generalitat de Catalunya, Barcelona, Spain

**Keywords:** evaluation, hospitality sector, secondhand smoke, smoking law, workplaces

## Abstract

**Background/objectives:**

A smoking law was passed by the Spanish Parliament in December 2005 and was enforced by 1 January 2006. The law bans smoking in all indoor workplaces but only in some hospitality venues, because owners are allowed to establish a smoking zone (venues > 100 m^2^) or to allow smoking without restrictions (venues < 100 m^2^). The objective of the study is to assess the impact of the Spanish smoking law on exposure to secondhand smoke (SHS) in enclosed workplaces, including hospitality venues.

**Materials and methods:**

The study design is a before-and-after evaluation. We studied workplaces and hospitality venues from eight different regions of Spain. We took repeated samples of vapor-phase nicotine concentration in 398 premises, including private offices (162), public administration offices (90), university premises (43), bars and restaurants (79), and discotheques and pubs (24).

**Results:**

In the follow-up period, SHS levels were markedly reduced in indoor offices. The median decrease in nicotine concentration ranged from 60.0% in public premises to 97.4% in private areas. Nicotine concentrations were also markedly reduced in bars and restaurants that became smoke-free (96.7%) and in the no-smoking zones of venues with separate spaces for smokers (88.9%). We found no significant changes in smoking zones or in premises allowing smoking, including discotheques and pubs.

**Conclusions:**

Overall, this study shows the positive impact of the law on reducing SHS in indoor workplaces. However, SHS was substantially reduced only in bars and restaurants that became smoke-free. Most hospitality workers continue to be exposed to very high levels of SHS. Therefore, a 100% smoke-free policy for all hospitality venues is required.

Exposure to secondhand smoke (SHS) has been recognized as a risk factor for a variety of diseases among exposed adults, including coronary heart disease and lung cancer. SHS exposure also causes respiratory symptoms and infections, asthma exacerbations, and an increased risk of sudden infant death syndrome in children (California Environmental Protection Agency 1997; International Agency for Research on Cancer 2004; U.S. Department of Health and Human Services 2006). Nonsmokers are known to be exposed to significant air pollution burdens from indoor smoking (Repace and Lowrey 1980). Current estimates suggest that SHS exposure might be responsible for as many as 19,400 annual deaths among nonsmokers in Europe alone (Smoke Free Partnership 2006). In Spain, a recent study has estimated a minimum of 1,228 annual deaths among non-smokers (López et al. 2007). In response to this growing evidence, smoke-free programs and policies have been widely promoted and implemented in public places and at the workplace. These initiatives have consistently shown clear benefits in several measures, including improving symptoms, self-reported health, and productivity (Borland et al. 1992; Chapman et al. 1999; Eisner et al. 1998).

In Europe, a growing number of countries have already adopted smoking regulations, although the overall approach to tobacco control differs (Joossens and Raw 2006; Spinney 2007). Most countries have banned smoking at the workplace, but there are large differences in policies focusing on the hospitality sector. The case of Italy is notable: Although a smoking ban in bars and restaurants that allowed smoking under several conditions was passed in 2005, in practice, only 1% of these venues has allowed smoking since the law came into force (Gorini et al. 2007).

Nowadays, there is widespread consensus that smoking control policies have represented a major step forward in protecting nonsmokers from SHS, thus producing a substantial gain in public health. However, some questions remain unanswered regarding the assessment of the impact of these policies in specific contexts. First, most studies have been carried out as pretest/post-test comparisons and have examined only short-term effects. Therefore, the mid- and long-term effects of these regulations remain unclear. Moreover, these studies have used a variety of indicators, including biomarkers (e.g., cotinine in saliva), airborne markers (e.g., nicotine or respiratory suspended particles), self-reported exposure, and health effects (e.g., respiratory symptoms). These indicators measure different dimensions of SHS exposure and have different validity. Among airborne markers, measurement of nicotine in vapor phase has been widely used because of its specificity, because SHS is the only source of nicotine in the air (Hammond 1993; Rothberg et al. 1998). This method has been used to evaluate the impact of smoking bans in the workplace (Heloma and Jaakkola 2003) and the hospitality sector (Gorini et al. 2005; Mulcahy et al. 2005) and has revealed significant changes after the implementation of new policies, even in a small number of venues.

Spain implemented a ban prohibiting smoking in all indoor workplaces in January 2006 (Fernandez 2006; Ministerio de Sanidad y Consumo 2005; Villalbí 2006). However, smoking is banned only in some hospitality venues: Venues > 100 m^2^ must either be smoke-free or have a smoking section (up to 30% of the total area) physically separated by a closed door and independently ventilated; venues < 100 m^2^ may decide to be smoke-free or to allow smoking without restrictions. Two years after the law enactment, only an estimated 10–20% of such venues have banned smoking (Martín-Luengo 2007).

As part of the evaluation of the impact of this law, we measured nicotine concentrations in the air as an indicator of SHS before the law was implemented (Sánchez-Martínez et al. 2007) and again 12 months after its implementation. In the present study we describe changes in nicotine concentrations in a variety of workplaces, including indoor offices and hospitality venues in Spain.

## Materials and Methods

We included offices in the public administration and private sectors, as well as universities and hospitality venues in the study at baseline (October–December 2005) and at follow-up 1 year later (October–December 2006) to assess changes in nicotine concentrations.

### Participant recruitment and sample size

We carried out this study in eight regions of Spain (Balearic Islands, Cantabria, Catalonia, Extremadura, Galicia, La Rioja, Madrid, and Valencia). In each region, we took 50 samples according to nonproportional quota sampling based on type of setting and size of hospitality venue (< 100 m^2^/> 100 m^2^). We selected the premises within each type of venue following a convenience sampling based on the feasibility and accessibility of the venues to the researchers.

We considered public administration offices to be offices in local, regional, and national administration. We selected one building from each level in each region and took four environmental samples from each building. In each region, we selected a university from which we took four air samples. In the private sector, we studied small (< 10 workers) and middle-size businesses (10–50 employees). In each region, we took six samples (from three different buildings) in small firms and six samples (from four different buildings) in middle-size firms. In the hospitality sector, we selected four restaurants > 100 m^2^, four restaurants < 100 m^2^, and four discotheques/pubs in each region, taking one sample in each venue. In restaurants that established separate areas for smokers and nonsmokers after the law came into force, we took samples from both areas. In public administration offices, universities, and middle-size private-sector offices, samplers were placed in the reception area, corridors, offices (desk positions), and toilets. In small offices in the private sector, samplers were placed in the reception area and offices (desk positions). In restaurants, samplers were placed in the main dining room.

We contacted the owners and managers of the selected facilities and venues either by telephone or by letter to explain the details of the study and to request permission. After obtaining permission, we arranged an appointment to place the samplers.

### Nicotine measures

We measured vapor-phase nicotine using SHS passive samplers, following the method described and validated by Hammond (1993) and used in previous studies of SHS assessment in Europe (Nebot et al. 2005). The samplers consisted of a 37-mm-diameter plastic cassette containing a filter treated with sodium bisulfate. These samplers were manipulated by instructed personnel according to a common protocol and placed in all the settings studied except pubs and discotheques for 7 days. The samplers that had to hang freely in the air were not placed within 1 m of an area where there was a regular smoker or where air did not circulate (e.g., a corner, under a shelf, or buried in curtains). In discotheques and pubs, where the expected concentration of nicotine was higher and operating hours were mostly at night, we took samples from personal monitors for short periods ranging between 4 and 5 hr. Personal samplers were clipped to a shirt collar or lapel, with the windscreen facing out, away from the clothes. They were carried out by volunteers.

For each sample, we recorded the following data: the sample’s code, region, setting, location, date and time of placement and removal, and smoking policy (smoking allowed, completely banned, or partially banned in separate zones). We recorded information on sampling area, sampling volume, and ventilation in each establishment to evaluate extreme or inconsistent values. We assigned samples with nicotine concentrations below the quantification limit a value of 0.01 μg/m^3^, corresponding to half the value of quantification limit for one sample exposed over a 1-week period. For quality control purposes, blank filters were placed within sampling filters (1 filter in 20) and all had nicotine concentrations below the quantification limit. Nicotine analysis was conducted at the Laboratory of the Public Health Agency of Barcelona, using the gas chromatography/ mass spectrometry method. The limit of quantification was 5 ng per filter. We estimated the time-weighted average nicotine concentration (micrograms per cubic meter) by dividing the amount of extracted nicotine by the volume of air sampled [estimated flow rate (24 mL/min) × the total number of minutes the filter had been exposed].

### Statistical analysis

We restricted the analysis to places where we took nicotine measurements both at baseline and follow-up (paired samples). Given the skewed distribution of nicotine concentration, we used median and interquartile ranges (IQRs) to describe the nicotine concentration by setting. We compared paired differences using the nonparametric Wilcoxon signed rank test. We used SPSS (version 12.0.1; SPSS, Inc., Chicago, IL, USA) for all the analyses.

## Results

Overall, we took 443 air samples at baseline in eight regions (autonomous communities) of Spain in the last trimester of 2005. We collected 398 samples (89.8%) again in the same venues at the 12-month follow-up. Table 1 shows the distribution by settings. According to the protocol, we took 162 samples in offices in the private sector, 90 in public administration offices (state, region, and city administration venues), 43 in university indoor premises, 79 in bars and restaurants, and 24 in discotheques and pubs.

Table 2 shows the change in nicotine concentration in workplaces other than hospitality venues at baseline and 12 months after the law was enacted. During the study period, there was a significant reduction in nicotine concentration, ranging from 60% in public administration to 97.4% in private sector offices. After the law, all medians were < 0.20 μg/m^3^.

Table 3 shows the changes in hospitality sector. The values are stratified according to the option taken after the law came into force. We found a significant reduction (96.7%) in places that became smoke-free. In venues allowing a smoking zone, we observed a similar reduction (88.9%) in no-smoking zones, whereas in smoking areas the median concentration increased slightly (37.2%). Venues allowing smoking had a nonsignificant reduction of 19.4%. Discotheques and pubs showed a nonsignificant reduction (from 33.3 to 15.1 μg/m^3^).

## Discussion

Overall, the results confirm the positive impact of the law in the indoor workplaces and hospitality premises that became smoke-free after the law. The median nicotine concentration decreased by 60.0% in public premises and by 97.4% in private workplaces. A major reduction (96.7%) also occurred in bars and restaurants that became smoke-free and in the no-smoking zones of venues where separate spaces were allowed (88.9%). In smoking zones and in premises allowing smoking, including discotheques and pubs, no significant changes occurred. As expected, the presence of SHS in bars allowing smoking, and in the smoking zones of those permitting separate zones, remained extremely high. Regarding differences in the proportions and nicotine levels between regions, stratifying by region, type of venue, and smoking regulation, the sample size in each stratum is too small to make statistically reliable comparisons.

The results of our study are consistent with those of previous studies that use nicotine in the air to evaluate the impact of smoking regulations. This method has proven to be both valid and sensitive and is therefore able to monitor changes in smoking policies with just a few samples. For example, seven discotheques and pubs were analyzed in Italy by Gorini et al. (2005), and 20 bars and pubs were studied in Ireland by Mulcahy et al. (2005). These studies found reductions in nicotine concentrations from 80% to 95% in bars that became smoke-free—percentages close to those found in our study.

Studies using other indicators have also detected changes. Some of these studies have used either other airborne markers such as particulate matter with aerodynamic diameters ≤ 2.5 μm (Goodman et al. 2007; Repace et al. 2006; Semple et al. 2007a; Valente et al. 2007) or biomarkers such as cotinine in saliva (Allwright et al. 2005; Semple et al. 2007b), and all have reported results very similar to ours. Furthermore, some of these studies used questionnaires to measure SHS exposure (Fong et al. 2006; Galán et al. 2007; Haw and Gruer 2007), although these studies cannot fully rule out some information bias.

A limitation common to many of the studies evaluating the impact of smoking policies is the short interval considered after the ban, in most cases only some weeks or months after the law was introduced. Only a few (Allwright et al. 2005; Goodman et al. 2007) have looked at the indicators 1 year after the law was enacted. As far as we know, only one study carried out in Italy (Gorini et al. 2008) evaluated the impact of the smoking policy 2 years after the implementation, showing an important decrease in nicotine concentrations even 2 years after the smoking ban. However, more studies are needed to rule out a possible “decay” effect of the smoking policies over the time.

This is the first study to show the impact of the Spanish law on SHS by using airborne markers and is among the few studies showing changes both in indoor workplaces and in hospitality sector venues. We have studied nearly 400 air samples, thus yielding by far the largest sample used in this kind of study.

In pubs and discotheques, filters were exposed for shorter periods (4–5 hr) than in other settings, which may have impaired comparability with other settings. However, we chose these time periods because typically these venues have most clients on the weekends and some are open only at this time. Therefore, exposing a filter for a whole week would have underestimated the real exposure. Because nicotine concentrations in these settings during working hours is very high (López et al. 2004, 2008; Nebot et al. 2005), a minimum of 4 hr is sufficient to detect the presence of nicotine above the minimum detection limit. We made measurements using the same procedure both sampling periods (before and after measurements), thus ensuring accurate estimation of changes in nicotine concentrations.

Another possible limitation could be the absence of a control group. However, control groups in evaluative public health research are not always necessary (or even possible) due to the complexities of the interventions evaluated (Victora et al. 2004). In this case, the characteristics of the law regarding the hospitality sector (i.e., permitting bars to choose between being smoke-free or non-smoke-free) allow the possibility of having two groups with different behaviors after the law, enabling comparison between hospitality premises that allow smoking and those that were smoke-free. Furthermore, the present study is a before-and-after study, in which comparison between the measurements taken before and after the law provide a valid and reliable estimate of the impact of the law.

Overall, this study shows the positive impact of the law in reducing SHS in indoor workplaces such as offices and provides a precise description of the law’s lack of effect in the hospitality venues that did not become smoke-free—a result that was largely anticipated by tobacco control advocates (Cordoba et al. 2006). In addition, this study shows the strong impact of smoke-free policies in the air of the few bars and restaurants banning smoking. In terms of public health, a large reduction in exposure has been achieved. However, workers in the hospitality sector remain exposed to very high levels of SHS, and therefore the situation cannot be considered satisfactory.

Assuming that approximately 80% (Martín-Luengo 2007) of hospitality workers in Spain (1,400,000) (Instituto Nacional de Estadística 2006) are still working in non-smoke-free hospitality venues and that the median nicotine concentration found in those venues in our study is associated with an excess lung cancer mortality risk of 98 per 100,000 (Repace and Lowrey 1993), the impact in terms of mortality burden could be as high as 1,000 deaths in hospitality-sector workers, if regularly exposed to this level of SHS for 40 years. Clearly, the results support a complete ban on smoking in all indoor places, including hospitality sector venues.

## Figures and Tables

**Table 1:** Settings studied and number of samples.

Setting studied	No. of samples at baseline	No. of paired samples 1 year after the law
Public administration	102	90
Local administration	30	27
Regional administration	44	42
National administration	28	21
Universities	43	43
Private sector	180	162
Small (< 10 workers)	53	49
Medium (10–50 workers)	127	113
Bars/restaurants	84	79
> 100 m^2^	46	45
< 100 m^2^	38	34
Discotheques/pubs	34	24
Total	443	398

**Table 2:** Median nicotine concentration (μg/m^3^) in workplaces at baseline and at the 12-month follow-up.

	Median nicotine concentration (IQR)			
Setting	Baseline	12-month follow-up	Percent variation	*p*-Value^a^
Public administration	0.20 (0.06–0.57)	0.08 (0.01–0.18)	−60.0	< 0.001
Local administration	0.46 (0.12–1.13)	0.13 (0.03–0.20)	−71.7	0.006
Regional administration	0.12 (0.06–0.38)	0.08 (0.01–0.20)	−33.3	0.020
National administration	0.20 (0.06–0.64)	0.05 (0.01–0.11)	−75.0	< 0.001
Universities	0.21 (0.08–0.50)	0.07 (0.01–0.15)	−66.7	< 0.001
Private sector	0.39 (0.07–1.29)	0.01 (0.01–0.16)	−97.4	< 0.001
Small (< 10 workers)	0.41 (0.05–1.40)	0.06 (0.01–0.18)	−85.4	< 0.001
Medium (10–50 workers)	0.39 (0.08–1.30)	0.01 (0.01–0.15)	−97.4	< 0.001

a Wilcoxon signed-rank test.

**Table 3:** Median nicotine concentration (μg/m^3^) in hospitality venues at baseline and at the 12-month follow-up.

	Median nicotine concentration (IQR)			
Setting	Baseline	12-month follow-up	Percent variation	*p*-Value^a^
Bars/restaurants				
Smoking banned^b^	2.71 (1.39–3.77)	0.09 (0.01–0.26)	−96.7	< 0.001
Smoking permitted throughout the premises^b^	7.07 (1.86–11.78)	5.70 (2.77–11.73)	−19.4	0.191
Smoking permitted in designated areas^b^				
Smoking area	5.58 (2.42–12.42)	8.89 (5.28–15.61)	37.2	0.075
Nonsmoking area	5.58 (2.42–12.42)	0.62 (0.34–1.40)	−88.9	0.036
Discotheques/pubs				
Smoking allowed^b^	33.31 (10.79–79.65)	15.06 (6.77–56.92)	−54.79	0.241

a Wilcoxon signed-rank test.

b Smoking regulation after the law; at baseline, smoking was permitted in all venues.
